# The propensity to adopt evidence-based practice among physical therapists

**DOI:** 10.1186/1472-6963-7-103

**Published:** 2007-07-05

**Authors:** Patricia H Bridges, Laura L Bierema, Thomas Valentine

**Affiliations:** 1 Division of Physical Therapy Department of Rehabilitation Medicine, Emory University School of Medicine, Emory University, Atlanta, Georgia, USA; 2 School of Leadership and Lifelong Learning, College of Education, University of Georgia, Athens, Georgia, USA; 3 School of Leadership and Lifelong Learning, College of Education, University of Georgia, Athens, Georgia, USA

## Abstract

**Background:**

Many authors, as well as the American Physical Therapy Association, advocate that physical therapists adopt practice patterns based on research evidence, known as evidence-based practice (EBP). At the same time, physical therapists should be capable of integrating EBP within the day-to-day practice of physical therapy. The purpose of this study was to determine the extent to which personal characteristics and the characteristics of the social system in the workplace influence the propensity of physical therapists to adopt EBP.

**Methods:**

The study used a 69 item mailed self-completion questionnaire. The questionnaire had four major sections. The first three sections were each drawn from a different theoretical framework and from different authors' work. The instrument was developed to capture the propensity of physical therapists to adopt EBP, characteristics of the social system in the workplace of physical therapists, personal characteristics of physical therapists, and selected demographic variables of physical therapists. The eligible population consisted of 3,897 physical therapists licensed by the state of Georgia in the United States of America. A random sample of 1320 potential participants was drawn.

**Results:**

939 questionnaires were returned for a response rate of 73%. 831 of the participants' questionnaires were useable and became the basis for the study. There was a moderate association between desire for learning (*r *= .36, *r*^2 ^= .13), highest degree held (*r *= .29, *r*^2 ^= .08), practicality (*r *= .27, *r*^2 ^= .07) and nonconformity (*r *= .24, *r*^2 ^= .06) and the propensity to adopt EBP. A negative correlation was found between age, years licensed and percentage of time in direct patient care. The findings demonstrated that the best three variables for predicting the propensity to adopt EBP in physical therapy were: desire for learning, highest degree held, and practicality.

**Conclusion:**

The study confirms there is no single factor to facilitate research evidence into day-to-day practice. Multiple practice change strategies will be needed to facilitate change in practice.

## Background

Health care practitioners today are increasingly urged to ensure that they are delivering care that is based on the best current research evidence, that is they are using evidence-based practice (EBP) to make clinical decisions. [1-5] Good clinical practice is shifting from practice based on tradition, expert opinion, unsystematic clinical experience, and intuition, to practice based on examinations and interventions that are backed by client centered research and other scientific studies. [6] EBP is a method for identifying, evaluating, and implementing good clinical data. Sackett, Strauss, Richardson, Rosenberg, and Haynes defined EBP as: "The integration of the best research evidence with clinical expertise and patient values". [7]

Adopting EBP may require change in practice, self-directed learning, and a favorable work environment. [8] The evidence-based health care practitioner must be able to understand the particular patient's circumstances, identify his/her gap in the knowledge for providing care based on the best research evidence, frame a question, find the best research evidence, and apply the findings to patient care. [9]

Many authors, as well as the American Physical Therapy Association (APTA) advocate that physical therapists adopt practice patterns based on research evidence. [2,4,5,10] Jette and colleagues report that members of the APTA generally have a positive view toward EBP. They found that physical therapists who were members of the APTA (*n *= 477) believe the use of research evidence in practice is necessary, that the literature is useful to them in clinical decision-making, and that the quality of patient care is superior when evidence is used. [6]

No matter how important EBP appears to be to the profession, the ultimate decision on whether or not EBP will be adopted and meaningfully used is going to be made by the clinician providing direct patient care in a practice setting that does or does not support EBP. It is not sufficient to generate and publish research evidence. [11] Examples of failure to integrate evidence into practice have been identified among physical therapists in Australia, England and the United States of America. [12,13]

The purpose of this study was to assess the factors that affect the propensity of physical therapists to adopt EBP through analysis of self report from physical therapists. Propensity to adopt EBP was defined for this study as the inclination physical therapists have to value research evidence from scientific studies when making clinical decisions. (See Table 1. for the scale of the items for the propensity to adopt evidence-based practice). The following research questions were investigated:

**Table 1:** Items measuring the propensity to adopt EBP from the factors affecting the propensity to adopt evidence-based practice survey

1.	In making clinical decisions, I value clinical experience more than scientific studies. (Reversed)*
3.	In making clinical decisions, seeking evidence from scientific studies makes a lot of sense to me.*
4.	In making clinical decisions, assessing the quality of the research evidence makes a lot of sense to me.*
7.	Clinical experience is the most reliable way to know what really works. (Reversed)*
9.	Patient care should be based where possible on scientific studies rather than the opinions of respected practitioners.*
10.	Critical appraisal of the literature and its relevance to the patient are not very practical in real patient care. (Reversed)*
12.	Practice guidelines for physical therapy should be based on evidence from scientific studies rather than consensus opinion.*
13.	Seeking relevant evidence from scientific studies is not very practical in real patient care. (Reversed)*

*Response Strongly Disagree < 1,2,3,4,5,6 > Strongly Agree

1. To what extent do personal characteristics predict the propensity to adopt EBP? (e.g. desire for learning, practicality, age) 2. To what extent do the characteristics of the social system in the workplace predict the propensity to adopt EBP? (e.g. empowerment, continuous learning) 3. To what extent do a combination of personal characteristics and the characteristics of the social system in the workplace predict the propensity to adopt EBP?

Little is known about how to get research evidence into practice. [12,14] The theoretical framework for this study was drawn from the adoption of innovation literature [15,16] and continuing medical education literature. [17,18] Factors that facilitate adoption of an innovation were studied by Rogers and Shoemaker [15] and Rogers. [16] Based on their work Cervero [17] developed a model that suggested that the type of change, the educational intervention, the personal characteristics of the learner, and the work environment in which the potential adopter was located affected adoption. This study tested two of those factors: personal characteristics of physical therapists and characteristics of the social system in which physical therapists worked. The study predicted the impact of personal characteristics of the individual physical therapist and the characteristics of the social system in the workplace on the propensity of physical therapists to adopt a particular innovation, EBP.

Several personal characteristics identified in the literature, self-directed learning, practicality and nonconformity [19-21] may facilitate the propensity to adopt EBP. Self-directed learning, a form of study in which individuals take responsibility for planning, conducting, and evaluating their learning activities, [19]has been linked to use of EBP. [7,20] Furthermore, adoption of EBP may require a physical therapist to depart from a traditional protocol that is practiced in their community or propagated by opinion leaders. [22]Clinicians will need to keep an open mind [23] and be willing to "diverge from common or previous practice" [21] In other words, they will need to be nonconformists. Nonconformity has been defined by Green, Gorenflo and Wyszewianski [21] as the "degree of comfort with engaging in clinical practices that are out of step with how others in the local community provide care or what opinion leaders recommend". [21] Finally, because lack of time has been identified as a barrier to the adoption of EBP [1] there was a notion that physical therapists who believe that they can use evidence-based guidelines and scientific studies without impacting productivity might be more likely to adopt EBP. Practicality has been defined for this study as the belief by physical therapists that evidence-based guidelines and scientific studies can be used to make clinical decisions in the day-to-day practice of physical therapy without interfering with productivity or the smooth and orderly flow of patients. [21]

The characteristics of the social system in the workplace may be important in determining the propensity to adopt EBP. Physical therapists similar to physicians learn from their work with patients, on teams with other health care professionals, and in dialogue with their colleagues. [18] A learning organization has the potential to provide opportunities such as critical debate on research evidence within the work environment that may facilitate the propensity to adopt EBP. [24,25]

## Methods

### Instrument, Validation and Limitations

Since an appropriate instrument that would gather desired data from the population to be studied was not located, a composite instrument was developed for the study. The instrument consisted of 69 questions and was organized into 4 sections. The instrument was developed to capture the propensity of physical therapists to adopt EBP, characteristics of the social system in the workplace of physical therapists, personal characteristics of physical therapists, and selected demographic variables of physical therapists. The central measure of the study was an 8 item scale measuring the propensity to adopt EBP which was adapted from a psychometric instrument by Green, Gorenflo, and Wyszewianski [26]. (Table 1.) A histogram was examined to ensure that the dependent variable, the propensity to adopt evidence based practice, was normally distributed. The histogram was judged to satisfy the assumption of normality. The scales for two related variables practicality and, nonconformity which collected data about personal characteristics were also adapted from the psychometric instrument. Each item in section 1 was scored using a Likert style 6 point scale where 1 = strongly disagree and 6 = strongly agree. Section 2 gathered the individual physical therapist characteristics of the social system in the workplace using the shortened version of the *Dimensions of the Learning Organization Questionnaire *(DLOQ). [27,28] Each item in section 2 was scored using a six point Likert style scale 1= almost never and 6 = almost always. Additional personal characteristic**s **were collected in section 3 using the shortened version of the *Self-directed Learning Readiness Scale for Nurse Education *(SDLRSNE). [29]Each item in section 3 was scored using a Likert style 6 point scale identical to the scale in section 1. Section 4 collected demographic information.

Section 1 was adapted from the psychometric instrument [26]to reflect the practice world of physical therapists. The section measured propensity to adopt EBP, practicality and nonconformity. An expert panel of five physical therapists modified the section for clarity and natural language of physical therapists and reviewed the section for validation of content validity for physical therapists. The experts had experience in a variety of practice settings for physical therapists and were chosen because they understood the practice of physical therapy.

Section 2 measured physical therapists' perception of the characteristics of the social system in the workplace using the shortened version of the *Dimensions of the Learning Organization *(DLOQ).[27,28] Evidence of the short form of the DLOQ's construct validity was established by Yang, Watkins and Marsick.[28]

Section 3 measured physical therapists' self-assessment as self-directed learners using a shortened version of the *Self-directed Learning Readiness Scale for Nurse Education *(SDLRSNE). [29] Fifty two items in 3 clusters: self-management, desire for learning and self-control were identified by a panel of experts in self-directed learning using the Delphi survey method as representing characteristics of a self-directed learner. [29] The instrument for this study used a shortened version of SDLRSNE including only the items that loaded at .45 criterion or higher on three factor analysis.

Table 2 depicts the distribution and reliability of scale variables for the dependent variable, propensity to adopt EBP and the independent variables, personal characteristics and characteristics of the social system in the work place. The scale mean and scale standard deviation were derived. Coefficient alpha was assessed to measure how well the items in each construct measured a single unidimensional variable. The coefficient alpha for individual variables ranged from .56 to .89.

**Table 2:** Distribution and reliability of the scale variables

	**n of items**	***M***	***SD***	***M of items***	**Coefficient Alpha**
***Dependent Variable***					
Propensity to adopt EBP using the evidence vs. experience scale	8	31.9	6.1	4.0	.83
					
***Personal Characteristics***					
Self-management	10	44.7	6.9	4.5	.87
Desire for learning	6	30.9	3.9	5.2	.85
Self-control	6	31.9	3.2	5.3	.80
Nonconformity	6	23.7	3.7	4.0	.56
Practicality	4	14.1	3.8	3.7	.66
					
***Characteristics of the Social System***					
Continuous learning	3	12.9	3.4	4.3	.79
Dialogue & inquiry	3	13.6	3.2	4.5	.87
Team learning	3	12.5	3.3	4.3	.81
Embedded systems	3	10.7	3.5	3.6	.79
Empowerment	3	11.5	3.4	3.8	.81
System connection	3	12.3	3.3	4.1	.80
Provide leadership	3	12.8	3.7	4.2	.89

### Participants

The potential participants consisted of 3,897 physical therapists who were licensed by the state of Georgia in the United States of America and were listed on a roster purchased from the state of Georgia. The goal was to create a sample large enough to generalize to the population of all physical therapists licensed by the state of Georgia. Based on Chassan's [30] recommendation and the advice of a statistician we selected 20 participants per varible as a desireable measure which would result in 660 potential participants. Twice that number were randomly selected from the list of potential participants to allow for nonrespondent problems and to ensure the stability of our analysis. 1320 names of potential participants were randomly selected using a computer generated software package *BCC Software*. 939 of the participants' questionnaires were returned for a response rate of 73%. 831 of the participants' questionnaires were useable and became the basis for the study.

Each therapist received an explanatory cover letter, survey instrument, a notice of *Research Information for the Participants *from the University of Georgia's Institutional Review Board, an incentive to participate, and a postage-paid return envelope. A follow-up reminder postcard was mailed 10 days later to all nonrespondents. Thirty-five days later all nonrespondents were contacted again by mail and sent another survey instrument, a different cover letter, Research Participants Information Sheet, another incentive to participate and a postage-paid return envelope. [31]

### Data analysis

*SPSS 11.5 *was used for all data analysis. Descriptive statistics, Pearson's correlation, a Spearman correlation, an independent t-test and stepwise multiple regression were conducted using *SPSS *statistical software package.

## Results

A total of 959 survey instruments were returned for a response rate of 73%. Of these, 831 of the survey instruments were useable. Surveys were discarded if the respondent indicated they were not currently working in the field of physical therapy or 16 or more of the items were not completed. Table 3 summarizes the demographic data. As can be seen the majority of the respondents were female (72.7%). Most respondents held an entry level bachelors degree (53.2%). The respondents ranged in age from 24 to 80, with a mean age of 39.4 years. The respondents' period of time as a licensed physical therapist ranged from two months to 55 years, with a mean of 13.4 years. Outpatient facilities and hospital settings were listed as the employment settings in which the physical therapists spent the majority of their time.

**Table 3:** Personal characteristics of study respondents (n = 831)

**Variable**	**Value**
Age	*M *= 39.4, *SD *= 9.5
Years as a licensed physical therapist	*M *= 13.4, *SD *= 9.8
Gender	
Female	72.7%
Male	27.3%
Race/Ethnicity	
White/Caucasian	86.1%
Asian	6.1%
Black/African American	4.6%
Hispanic	1.1%
Native Hawaiian and other Pacific Islander	0.4%
American Indian/Alaska Native	0.1%
Other	1.7%
Highest degree held	
Bachelor's	53.2%
Master's	42.4%
Doctorate in Physical Therapy	1.4%
Other Doctorate	1.8%
Other	1.2%
Employment setting	
Outpatient	41%
Hospital	20.8%
Home Health Agency	12%
Skilled Nursing Facility/Extended Care/Assisted Living Facility	7.2%
Acute Rehab or Sub-acute Rehab Hospital	4.3%
School System	3.6%
Academic Institution	1.8%
Other	3.7%
Multiple listings	5.4%
Percentage of time respondents spend performing selected activities	
Direct Patient Care	*M *= 77.5%, *SD *= 22.6
Administration	*M *= 13.4%, *SD *= 18.5
Education	*M *= 5.7%, *SD *= 10.6
Research	*M *= 1.4%, *SD *= 5.9
Other	*M *= 2%, *SD *= 6.6

In response to the first research question: "To what extent do personal characteristics predict the propensity to adopt evidence-based practice?" Most of the personal characteristics were predictors of the propensity to adopt EBP. Out of the nine variables tested, eight were statistically significant at the .05 level or better. The strongest correlation was a subscale of self-directed learning, desire for learning (*r *= .36, *r*^2 ^= .13). The other two subscales of self-directed learning, self-control (*r *= .18, *r*^2 ^= .03), and self-management (*r *= .09, *r*^2 ^= .01) contributed to the variance in the propensity to adopt EBP. Practicality (*r *= .27, *r*^2 ^= .07) and nonconformity (*r *= .24, *r*^2^= .06) contributed moderately to the variance in the propensity to adopt EBP. When variables were measured at the interval level as were all scale variables, a Pearson's correlation was run. When the variable was ordinal which was the case with one variable, highest degree held, a Spearman correlation was run. The Spearman correlation test demonstrated a significant relationship at the .05 level between highest degree held and the propensity to adopt EBP (*r*_s _= .29). In one case where the predictor variable was dichotomous (gender) a t-test was run. In comparing the scales based on gender the means were very close.

In response to the second research question: "To what extent do the characteristics of the social system in the workplace predict the propensity to adopt evidence-based practice?" Continuous learning, empowerment, and system connection were significant at the .03 level or better. Empowerment (*r *= .11, *r*^2 ^= .01), continuous learning (*r *= .08, *r*^2 ^= .01), and system connection (*r* = .08, *r*^2 ^= .01) made minimum contributions to the observed variance in the propensity to adopt EBP. Table 4 summarizes variables predicting the propensity to adopt EBP.

**Table 4:** Pearson's correlation and spearman correlation of variables predicting the propensity to adopt EBP

***Variable***	***r***	***r*^*2*^**	***p***
***Personal Characteristics***			
Desire for learning	.36	.13	.001
Practicality	.27	.07	.001
Nonconformity	.24	.06	.001
Self-control	.18	.03	.001
Years licensed as a physical therapist	-.10	.01	.002
Self-management	.09	.01	.006
Age	-.07	.01	.026
Percentage of time spent in direct patient care	-.07	.01	.025
Percentage of time spent in administration	-.04	.00	.114
Highest degree held	.29*	----	.001*
			
***Characteristics of the Social System***			
Empowerment	.11	.01	.001
Continuous learning	.08	.01	.015
System connection	.08	.01	.012
Team learning	.06	.003	.062
Provide leadership	.04	.002	.109
Embedded systems	.04	.002	.123
Dialogue & inquiry	.03	.001	.233

*Spearman Correlation

In response to the third research question: "To what extent do a combination of personal characteristics and the characteristics of the social system in the workplace predict the propensity to adopt evidence-based practice?" The forward selection method was used to find the best one variable model, the best two variable model, and the best three variable model. Desire for learning significantly predicted the propensity to adopt EBP (*P* =.05, *R*^2 ^= .14). The best two variable model consisted of desire for learning and highest degree held. Adding a second variable accounted for 19% of the observed variance of the propensity to adopt EBP (*P *= .05, *R*^2 ^= .19). The three variable model consisted of desire for learning, highest degree held, and practicality. Adding the third variable accounted for 23% of the variance in the propensity to adopt EBP (*P *= .05, *R*^2^= .23). Table 5 contains the results of the analysis.

**Table 5:** Best one, two, and three variable models

Model	Model *R*^2^	Predictor	Beta
One variable model	.14	Desire for learning	.369
Two variable model	.19	Desire for learning	.333
	.19	Highest degree held	.241
Three variable model	.23	Desire for learning	.282
	.23	Highest degree held	.236
	.23	Practicality	.187

Note: The F statistic for each of the 3 models was significant at the .05 level. The t statistic for each parameter was significant at the .05 level.

## Discussion

Incorporating EBP will depend on whether or not the individual physical therapist providing direct patient care has the propensity to integrate the best current research evidence available with patient values and clinical experience [7] and then apply the research evidence to the prevention, assessment and intervention of physical therapy problems across the continuum of care. [10] The purpose of this study was to identify the factors that affect the propensity of physical therapist to adopt EBP. The study demonstrated that multiple factors influence physical therapists' propensity to adopt EBP. Personal characteristics contributed significantly to the variance in the propensity to adopt EBP.

### Self-directed Learning

Lifelong self directed learning is essential in the context of a rapidly growing and changing body of knowledge in health care. [32,33] The study confirms that desire for learning, a component of self-directed learning, accounts for a modest proportion of the variance in the propensity to adopt EBP. The information seeking behavior common to both self-directed learning and EBP may account for the association. A fully self-directed learner is one who has learned to think critically. [29,34]It is the critical reflection on practice that forms the basis for questions that lead to locating the best current research evidence.[35] Developing a question also requires other behaviors identified as characteristics of the self-directed learner in the literature: initiative, [19,29] self-discipline, [19,33]and the ability to develop and set goals. [19] Initiative and self-discipline were consistent with the behaviors described in the self-management scale, which emerged as a minimum predictor of the propensity to adopt EBP. Goal setting was addressed in two of the items in the self-control scale, which emerged as a minimum predictor of the propensity to adopt EBP.

### Highest Degree Held

This study provides empirical evidence that the higher the degree of education physical therapists obtain the more likely they are to demonstrate the propensity to adopt EBP. This finding is consistent with previous research by Warren and Pierson [36]who found that physical therapy students with a master's degree demonstrated a more positive attitude toward research than baccalaureate students.

### Age, Years Licensed as a Physical Therapist, and Time Spent in Direct Patient Care

Age and years licensed as a physical therapist were negatively correlated with the propensity to adopt EBP. When we examined age and years licensed with the predictor highest degree held, we extrapolated that physical therapists who were older and have been licensed longer were less likely to demonstrate the propensity to adopt EBP because they may not understand how to access the scientific literature. Time spent in direct patient care was negatively correlated with the propensity to adopt EBP. The negative correlation is noteworthy. Based on the findings of this study, physical therapists who are providing direct patient care value clinical experience and authority over scientific studies when making clinical decisions. This suggests direct patient care continues to be based on clinical experience and authority. Reaching the provider of direct patient care is imperative if the paradigm is going to shift to practice based on "the integration of the best research evidence with clinical experience and patient values." [7]

### Practicality

Numerous authors have cited lack of time as a barrier to the adoption of EBP. [1,37,38] It is therefore not surprising that those physical therapists who reported that they agreed that evidence-based guidelines and scientific studies can be used to make clinical decisions in the day-to-day practice of physical therapy without interfering with productivity or the smooth and orderly flow of patients [21] demonstrated the propensity to adopt EBP. This finding suggests that research evidence needs to be easy to access, time efficient, and relevant to practice so that productivity and the smooth orderly flow of patients will not be adversely affected. Linking evidence to workflow so that physical therapists can act on it in a time efficient manner is essential. [39]Research evidence needs to be available at the point of care so that clinical questions can be answered quickly. [40]

### Nonconformity

The study demonstrated there was a relationship between physical therapists who view themselves as engaging in clinical practices that are out of step with how others in the local community provide care or what opinion leaders recommend, nonconformist, [21] and the propensity to adopt EBP. We speculated that as physical therapists have moved toward autonomous practice in the United States and thereby perceived themselves as having the capability, ability, and responsibility to exercise professional judgment, they concomitantly demonstrated a propensity to value research evidence over experience and authority when making clinical decisions. EBP may require a physical therapist to depart from the usual and ordinary care that has been provided by other physical therapists in the facility in order to provide the right treatment for the right patient based on the best current research available. [41]

### Multivariate findings

The variables desire for learning, practicality, and highest degree combined account for a moderate proportion of the observed variance in the propensity to adopt EBP. This combination of variables out performed any single variable predicting the propensity to adopt EBP. This finding reveals the fact that a combination of factors works synergistically to foster the propensity to adoption of EBP.

### Limitations

Despite the theoretical integrity of these predictors we have limited explanatory power. The predictor variables employed in this study were selected based on a review of the literature. However, as with any study we studied only a finite number of variables. There were many variables that could have been considered including: skills in critically appraising the literature, computer access, knowledge of online databases, amount of time available to research evidence, presence of weekly case studies, or presence of a journal club in the facility or the community.

Another limitation deals with the sample. Because the sample frame only covered physical therapists licensed by the state of Georgia in the United States of America generalization to other groups must be handled by logical rather than statistical inference.

### Application

This study has a number of implications for the practice of physical therapy and other health care practices. In this study desire for learning, a component of self-directed learning, accounts for a modest proportion of the variance in the propensity to adopt EBP. Adults learn from experience and from problems in their everyday life. [19] Each day questions arise in the practice of health care that go unanswered. [35] It is in the day-to-day practice that continuing education should be taking place. Cervero, an expert in continuing education of medical professionals, has proposed that if "physicians are going to make good clinical judgments they need to learn from their experience in the swamp of practice."[42]The same is true for all health care practitioners. EBP harks back to the natural teaching method described by Osler. [43] The patient is the source of the health care practitioner's inquiry. To maximize the benefit of self-directed learning health care practitioners will need to reflect on practice and develop a process of questioning. It is this critical reflection on practice that forms the basis for questions that lead to locating the best current research evidence on interventions. [35] Reflecting on practice illustrates to members of the health care team how the ordinary challenges of work can be converted into opportunities for learning. [24] To further foster a desire for learning and encourage the use of EBP opportunities should be made available to provide health care practitioners the leisure to "reflect on their reflection in action" [42] in clinical decision making in the presence of colleagues. In other words there should be a forum where there is critical debate of the treatment interventions based on the best current research evidence available. These communities of practice [44] can take the form of weekly journal clubs or case studies. Exploiting the problems that arise in day-to-day practice of the health care practitioners by reflecting on practice, converting the patient problems to questions and reflecting on actions with colleagues develops habits of practice that are consistent with facilitating self-directed learning. [38,45,46]

Practicality accounted for a moderate proportion of the variance in the propensity to adopt EBP among physical therapists. It is imperative that the health care professions, adult educators, change agents and department managers investigate ways to make EBP easy to access, time efficient, and relevant to practice so that productivity and the smooth orderly flow of patients will not be adversely affected. Research evidence needs to be linked to workflow so that health care practitioners can act on it in a time efficient manner. [39]The best current research evidence for treatment interventions needs to be readily available electronically in the same place and at the same time that a health care professional is making a clinical decision so that the right treatment intervention is provided for the right patient in a timely manner.

## Conclusion

In conclusion, this study has begun the work of identifying personal characteristics that will foster the propensity to adopt EBP. Three predictors, desire for learning, highest degree held, and practicality accounted for a moderate proportion of the variance of the propensity to adopt EBP. Nonconformity was associated with the propensity to adopt EBP. Age, years licensed as a physical therapist and time spent in direct patient care were negatively correlated with the propensity to adopt EBP. The findings of the study suggest the characteristics of the social system made a minimum contribution to the observed variance in the propensity to adopt EBP.

Much can be learned from the investigation of the propensity to adopt evidence based practice among physical therapists that can be applied to other health professions. The health professions, adult educators, change agents and department managers need to exploit these findings when developing strategies to foster the adoption of EBP. Suggestions were made for fostering self-directed learning by using the natural teaching method of reflecting on the problems at the point of care. Furthermore, evidence-based practice needs to be made user friendly so that it does not interfere with the smooth and orderly flow of patients. Future studies should focus on the availability of evidence and ready access to evidence as factors which affect the propensity to adopt EBP.

## Competing interests

The author(s) declare that they have no competing interests.

## Authors' contributions

PHB conceived the study, participated in the design of the study, participated in the development of the instrument, coordinated the study, and drafted the manuscript.

LLB participated in the design of the study and reviewed the manuscript.

TV participated in the design of the study, participated in the development of the instrument and performed the statistical analysis.

## Pre-publication history

The pre-publication history for this paper can be accessed here:


